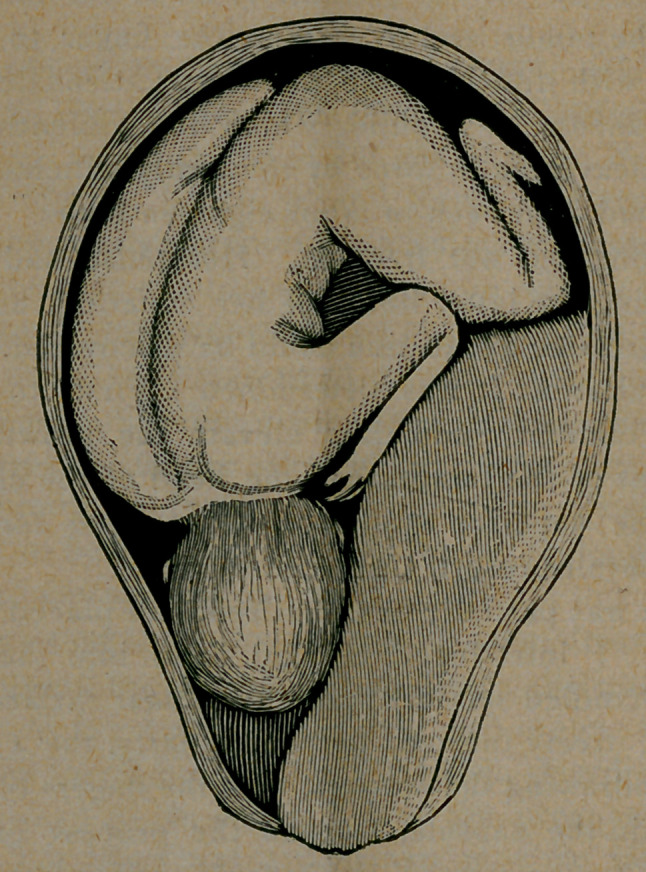# Interstitial Uterine Fibro-Myoma Complicating Delivery

**Published:** 1887-05

**Authors:** E. J. Beall

**Affiliations:** Fort Worth, Texas


DANIEL'S
### Texas Medical Journal.

PUBLISHED MONTHLY.— SUBSCRIPTION $2.00 A YEAR.

Vol. 2. AUSTIN, MAY, 1887. No. 11.

### ORIGINAL ARTICLES.

CONTRIBUTED EXCLUSIVELY TO THIS JOURNAL.

*The Articles in this Department are accepted and published with the understanding that we are not responsible for, nor do we indorse the views and opinions of the writers by so doing.*

### INTERSTITIAL UTERINE FIBRO-MYOMA COMPLICATING DELIVERY.

*By E. J. Beall, M. D., Fort Worth, Texas.*

For Daniel's Texas Medical Journal.

UTERINE fibroids, at their commencement, are interstitial. They may remain interstitial, or may become sub-peritoneal, or sub-mucous. Either of these formations may occur independently of pregnancy and parturition, or may complicate that process. Other tumors beside the fibroid may originate in connection with the uterus, its appendages, and the cavity which surrounds it; and may exist with or without the concurrence of pregnancy.

This brief paper is intended to bring before the profession a case of interstitial fibroid, complicating labor; and the remarks herein will be confined to the variety of tumor, in relation to which that designation obtains.

Interstitial fibroids are of quite common existence. The *contrary*

is true when conception has occurred. Now and then, however, cases arise in which conception took place and full term is reached; others in which circumstances require interference before or after the viable period, in order to preserve life.

Interstitial fibroids may occupy any portion of the uterus, but are more common in connection with the body than the cervix, (1) Munde.

If small, conception may occur; if large, not so likely to do so. When they exist, and pregnancy takes place, with the physiological development of the uterus there is a *pari passu* growth of the fibroid; hence, at full term, an interstitial fibroid may have assumed large proportions with no symptom appearing to indicate the presence prior to conception, or during gestation.

When pregnancy has occurred, and an interstitial fibroid was at its inception, or at the beginning of its development, the location it may occupy may interfere with normal development of the fœtus and uterus, jeopardizing the safety of the former, mechanically, as well as determining an irregular nutrition of the latter, thus endangering the life of both fœtus and mother prior to, and when the normal term of gestation is reached.

During development the fœtus may become deformed by pressure of the growing myoma, deflecting limbs, disfiguring the head, or any portion of the body it may encroach upon; may, perhaps, crowd the child into a preternatural position, retarding its growth and add very much to the discomfort of the pregnant and parturient woman. The placental attachment may be interfered with, producing, perhaps, atrophy, or detachment with the necessary bleeding that would follow; crowding downwards infringe upon the bladder, vagina, perhaps bowels, and thus mechanically endanger the comfort of the subject. These and other ills may occur to child and mother prior to the emptying of the uterus. After delivery, natural or artificial, other dangers beset the woman and her offspring. Peradventure, both may perish—victims of nature's efforts for delivery, or the means essayed for relief by the obstetric surgeon. This subject will be further presented when referring to the plans pursued in the past for relief in such cases.

February 21, 1887, I was summoned by telegram to an adjacent county by Drs. Hall, Pickett and Menafee, to see, with them, Mrs. Haskew, who was then in labor, the labor complicated with a large interstitial fibroid. Mrs. H. was forty-four years old, married ten years, in labor with fourth child. I arrived at the scene at 10 o'clock p. m. The patient then had been in labor eighteen hours; the amniotic fluid already evacuated; os uteri dilated to two-thirds its capacity, and was occupied by the large, hard, unyielding tumor, as shown in the cut. It was possible to make out the cervical attachment of the tumor, and by forcing the finger to the left (her right) to find, with difficulty, the presenting attenuated corrugated caput succedaneum of a child, which the violently contracting uterus was endeavoring to force by the firmly adherent mass. The patient, as stated, had been in labor eighteen hours. The contractions were strong, but not frequent. Her pulse had increased in frequency during the latter hours of her suffering, and other evidence of progressive exhaustion could be discovered.

Upon consultation, it was determined: First, to endeavor to enucleate the tumor. Second, to elevate the growth. Third, to incise sufficient of the tumor, that the head of the child might be engaged in the superior strait, or be reached for craniotomy. Fourth, as a dernier resort, Cæsarean section.

The woman was partially anæsthetized, and placed in the lithotomy position. I introduced my left hand into the vagina, and, guiding long handled scissors upon the palmar surface of the intra-vaginal hand, freely incised, perpendicularly, the capsule of the fibroid. Withdrawing the scissors, with the fingers of the hand introduced within the capsule, I began the process of enucleation. I followed the tumor upward, the capsule covering the dorsal, and the palmar surface approximated to the tumor, until my arm was hidden within the soft parts nearly to the elbow. It was only by this degree of penetration I could reach the superior limit of the tumor. The left hand was now withdrawn, the right inserted, and the enucleation completed upon the opposite side of the tumor. This done, during the presence of a contraction, I grasped the fibroid with my hand, and, by twisting, liberated the few apex attachments that I could not readily reach during the enucleating process, and withdrew the body from the uterus. As the tumor was withdrawn during a "pain," I kept the left hand upon the the outside of the uterus to stimulate contraction, a precaution against hemorrhage. Hitherto I had used it to immobilize the tumor and uterus while the enucleation was in progress. Immediately before the delivery of the tumor, one fluid drachm of fluid extract of ergot was administered hypodermically, believing that the child would soon follow through a vagina so greatly dilated, and that the good effect of that agent might be displayed as an aid to guard against post partem hæmorrhage.

The tumor weighed two pounds and thirteen ounces. The manipulation and subsequent treatment was after the strict order that should guard against the septic infection as was practicable under the circumstances.

The pain succeeding the one in which the tumor was delivered engaged the child as in a natural labor; and recurring pains soon brought to view a female child weighing fully ten pounds. There was some difficulty in inducing respiration; but after the lapse of fifteen or twenty minutes, the babe was as well as could be desired.

The caput succedaneum was developed at the side of the vertex; the fontanel somewhat displaced from median line indicating and corresponding to the pressure of the fibroid during the latter months of gestation.

The uterus contracted well; the placenta was immediately expressed; and no greater amount of flooding occurred than is usual in the "relaxed uteri" of women who have reached the age of the patient herein considered.

The health of this woman had been fairly good for a number of years. Her periods prior to the last conception were little, or no more menor or metrorrhagic than was her habit in the earlier years of her married life. The physician, Dr. Pickett, who delivered her two years prior to her last confinement, was present, and asserted that he was not, nor was any one else, cognizant of any symptom pointing to the existence of a tumor connected with the uterus until taken in the labor just detailed. Although we may not know positively, we may reasonably presume, the fibroid complicating this full term labor was very small, if, indeed, it existed when conception occurred. That the growth was rapid during gestation its size and attachments clearly indicate, there having been no evidence of the existence of the tumor prior to her confinement.

It is now the purpose of the writer to present such cases of interstitial fibroids complicating labor as limited time and facilities have enabled him to collect; present abstracts that shall indicate plan of treatment pursued and result, with the view of aiding future observers in arriving at the best mode of proceedure under such circumstances. It is true such cases are rare; but having once occurred, will occur again, and to whom no one can know.

It is due the profession that such cases be made professional property. Our experience really belongs, and should be contributed, to a general fund of professional knowledge. Though the results be good or bad, in either event a sign board will be erected to guide future workers in the obstetric art.

The pertinent question in relation to interstitial myoma is, how and when shall we interfere? Dr. Playfair claims that only small fibroids complicating delivery can be enucleated. Other practitioners have disproved the assertion by the successful completion and issue of such cases. At the time Dr. P. perpetrated such views before the London Obstetrical Society, Dr. Braxton Hicks challenged the position assumed, and gave the history of a successful case coming under his observation. Still others have disproved Dr. Playfair's position, notably, Munde, Danyan, Grimsdale,

Wallace, Depaul, Schroeder, Langenbeck, Keating, Fry, and my own successful case.

In cases to which enucleation is applicable, and I think they are more numerous than have hitherto been so considered, the results in the few cases thus treated throw a damper over other procedures and an analysis of cases will indubitably establish this fact.

If a case is presented, in which the tumor can be elevated, and labor completed by the natural powers of the parturient woman, or the interference of art, the tumor still remains, and except in the few cases in which, during the process of uterine involution, fatty degeneration likewise ensues—as demonstrated in the progressive atrophy of the fibroma—the tumor still remains, a matter for subsequent treatment with the attendent dangers inseparable connected therewith. Brown and others have reported such cases. An examination of a critical character will indicate that the hope for post partem disappearance of the growth is the exception. The rule being that the tumor will remain with its tendencies to hemorrhage, to the complication of subsequent pregnancies and other ill results indefinitely. Cases are of record which sustain this position.

The parts at delivery are lubricated, and in other ways prepared by nature for child expulsion. There is a *vis a tergo*, as it were, in the then existing uterine contractions, which favor more than at other times the incision and enucleation of such growths from the cervix and lower uterine segments. The results of enucleation in the twelve or thirteen cases in which that plan was adopted have been so good, to both, to mother and child, that the imperative duty of every practitioner is, when he shall meet with interstitial fibroids, to endeavor to make a decided and thorough effort to manage such cases by the plan of enucleation. If successful, the child is often times spared destructive means in order to relieve the mother. For in the event the tumor can be elevated, the inducement will then present itself to the surgeon, to attempt an artificial delivery by version, the forceps, or craniotomy. If one shall entertain the suggestion to reduce the tumor by incision of the more dependent portion in order that the head may engage in the straits, the same arguments against the elevation of the tumor hold good, and to greater degree of force ; for then other dangers are added ; more or less increase in blood loss, and exaggerated chances for septic infection. The mortality attending the Cæsarean section, in which the tumor is left behind, the resort to Porro's operation, the plan suggested by Bricksy, must ever remain as the dernier resort, held in reserve when decided efforts have been first made to enucleate and failure has resulted. Dr. Thomas once did a Cæsarean section for a myoma complicating delivery. He says: "That since the construction of his saw-spoon, as an aid in difficult enucleation future operators, with that instrument, may be enabled to succeed in enucleation when hitherto severer measures would have been instituted."

When an enucleation is being practiced the hand is the best means for the accomplishment of our purpose; when, however, fibrous bands exist that resist the efforts of the fingers, then the serrated spoon of Thomas, referred to above, may be brought to good use.

I believe, in cases where the fibroid is easily lifted, or where the attachments are high and labor terminates, the time then exists for the enucleation of the tumor. I think it better then when the uterine cavity is patent, an easier operation will be presented, than at a future time, and fraught with much less danger to the woman.

It can be inferred from the tenor of this article, that a myoma may complicate gestation, and no symptom occur to indicate its existence. When, however, the opposite is the case, symptoms point to the presence of the tumor,—when shall the surgeon act, taking into consideration, the well being of both mother and offspring? This question, I think, should be determined in each individual case. Let the case be a "law unto itself." If bleeding has not occurred, when the tumor has been discovered, or is not excessive, can be held in abeyance. If no threatening symptoms exist, it would, perhaps, be well to await the period of viability; and, if possible, full term. If success would have followed the induction of premature labor peradventure at full term, the condition would not have so changed as to render like success unattainable.

This paper, more than half of which has been written at odd moments, after the Association was in session, is not as complete as I wished. Many points connected with the interesting subject I leave untouched. I cease writing with the hope, that should any member of the "Texas State Medical Association" ever encounter the rare condition of a fibro-myoma complicating delivery, that decided, and intelligent efforts shall be essayed for the enucleation of the tumor. And I farther hope, that results may prove as felicitous, as they were in the case occuring to myself,—for now a mother lives six weeks removed from the terrible ordeal through which she passed; well and gratified in the enjoyments and pleasures growing out of the sweet communion of a dear babe, whose life was once in the "balance" with her own.

NOTE.—It is intimated in the body of the paper, that a table of cases of interstitial fibroids, treated by enucleation, would be prepared. Want of time prevented my making the abstracts intended.

[NOTE.—The above paper was promised us for our April number, and was in preparation, but the author was called away and did not complete it. A subsequent thought was to send it to the Association meeting, but it arrived after adjournment, and is produced here by authority of the writer.]

## Figures and Tables

**Figure f1:**